# Emerging trends in immunotherapy for pediatric sarcomas

**DOI:** 10.1186/s13045-019-0756-z

**Published:** 2019-07-16

**Authors:** Kyle A. Dyson, Brian D. Stover, Adam Grippin, Hector R. Mendez-Gomez, Joanne Lagmay, Duane A. Mitchell, Elias J. Sayour

**Affiliations:** 1 Preston A. Wells, Jr. Center for Brain Tumor Therapy, University of Florida Brain Tumor Immunotherapy Program, Lillian S. Wells Department of Neurosurgery, McKnight Brain Institute, University of Florida, 1149 South Newell Drive, Gainesville, FL 32611 USA; 2 Division of Pediatric Hematology Oncology, Department of Pediatrics, University of Florida, PO Box 100298, Gainesville, FL 32610 USA

**Keywords:** Immunotherapy, Pediatric sarcoma, Osteosarcoma, Ewing’s sarcoma, Rhabdomyosarcoma, Cancer vaccines, Adoptive cellular therapy, CAR T cell therapy, Checkpoint blockade

## Abstract

While promising, immunotherapy has yet to be fully unlocked for the preponderance of cancers where conventional chemoradiation reigns. This remains particularly evident in pediatric sarcomas where standard of care has not appreciably changed in decades. Importantly, pediatric bone sarcomas, like osteosarcoma and Ewing’s sarcoma, possess unique tumor microenvironments driven by distinct molecular features, as do rhabdomyosarcomas and soft tissue sarcomas. A better understanding of each malignancy’s biology, heterogeneity, and tumor microenvironment may lend new insights toward immunotherapeutic targets in novel platform technologies for cancer vaccines and adoptive cellular therapy. These advances may pave the way toward new treatments requisite for pediatric sarcomas and patients in need of new therapies.

## Introduction

Immunotherapy has heralded much promise for cancer as demonstrated by rapidly increasing numbers of FDA-approved therapies for malignancies resistant to conventional therapies. Despite immunotherapy’s promise, its benefits have yet to be unlocked for the preponderance of cancers where conventional chemoradiation predominates. This is particularly noteworthy for pediatric sarcomas where the standard of care has not appreciably changed in several decades.

### Pediatric vs adult sarcomas

The biologic underpinnings for pediatric sarcomas differ from what is traditionally understood of their adult counterparts. In children and adolescents, the most common sarcomas include osteosarcoma (OS), Ewing sarcoma (EWS), rhabdomyosarcoma (RMS), and desmoplastic small round cell tumors, while other sarcomas, such as leiomyosarcomas and liposarcomas, typically present in older patients. Sarcoma histologies can behave differently depending on the age of the patient. For example, adult fibrosarcoma and EWS are generally more aggressive than their pediatric forms [1]. In contrast with pediatric sarcomas, adult malignancies are predisposed to increased mutational loads, while oncogenic gene fusions are commonly detected in pediatric sarcomas [2]. Although increased mutational loads may be co-opted for immunotherapeutic benefit, the childhood immune system is expected to be more malleable. Differences in the immune systems of children and adults have important practical implications for cancer immunotherapy. The pediatric immune system is more robust and cellular relative to older patients; this is corroborated in part by quick immunologic recoveries in children receiving chemotherapy. Thus, in addition to unique factors such as a patient's HLA haplotype, distinctions in both immune function and sarcoma biology between adult and pediatric patients will affect response to immunotherapy. Before immunotherapy can be fully leveraged for pediatric sarcomas, a better understanding of their tumor microenvironment (TME) is essential. This understanding, in juxtaposition with identification of new therapeutic immune targets, may be co-opted to slowly remold the intratumoral milieu, enabling immunotherapy to benefit more patients [1, 3].

## Tumor immune microenvironment

**Fig. 1:**
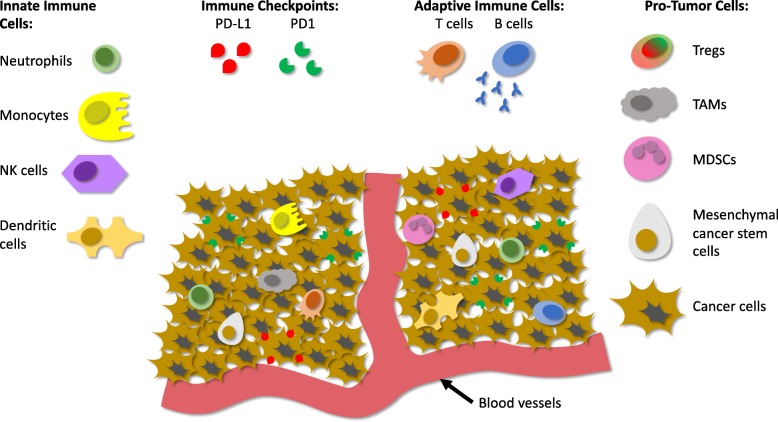
Depiction of the Sarcoma Tumor Microenvironment

The sarcoma immune microenvironment exists as a network of innate (i.e., neutrophils, macrophages, monocytes, natural killer (NK) cells, and antigen presenting cells) and adaptive immune cells (i.e., B and T lymphocytes) (Fig. 1). While immunologic response against tumor targets can be edited out in the early phases of oncogenesis [4], in OS (the most common malignant bone tumor in children), the presence or absence of these immune cells has been linked to prognosis [5]. Notably, sarcoma samples devoid of immune infiltrates display increased metastases and poor overall survival [5]. Immunologic markers of response, both within the systemic and intratumoral microenvironments, may be necessary to better understand the pediatric sarcoma immune system for development of targeted immunotherapies.

### Lymphocytes

OS can be enriched in CD8^+^ T lymphocytes (with low CD4^+^ T cells), CD20^+^ B lymphocytes, and CD117^+^ mast cells [6]. The pretreatment neutrophil-to-lymphocyte ratio (NLR) and absolute lymphocyte count (ALC) recovery in pediatric OS and RMS patients have been evaluated as early prognostic indicators for sarcoma risk stratification [7]. An NLR > 2 and decreased ALC recovery (15 days post-treatment) were associated with increased hazard ratios in pediatric sacroma patients, suggesting that cytotoxic therapy plays an important role in depleting regulatory lymphocyte populations (i.e., FoxP3+ Tregs) and enabling effector lymphocyte populations to engage shed cancer antigens [7]. For other cancers like EWS, the immune system may be more senescent due to a lack of oncologic drivers outside of the EWS translocations. Interestingly, in EWS, the MHC class I molecule HLA-G expressed locally on tumor cells and tumor-infiltrating lymphocytes (TILs) has been studied as an immune regulator. This molecule is associated with an increase in number of TILs and it has been theorized that EWS cells upregulate HLA-G as a mediator of immune escape [8]. Future pediatric sarcoma TME studies should assess not only TIL numbers and percentages, but also contributions from their respective effector vs regulatory compartments. In OS, for example, studies have shown that higher CD8^+^/FOXP3^+^ ratios correlate with improved survival and a better prognosis [9]. A better understanding of cellular phenotype, function, and cross-talk with systemic immunity will be requisite to not only understand the TME of pediatric sarcomas but also skew their peripheral response toward effector populations.

### Myeloid cells in the TME

In addition to lymphoid populations, effector and regulatory myeloid populations also exist in the sarcoma TME, including tumor-associated macrophages (TAMs), where they contribute to the regulation of local immunity and exert pleotropic effects on angiogenesis and tumor cell migration [10]. New techniques have been developed to quantify TAMs for assistance in risk stratification of sarcomas, including MRI imaging following administration of ferumoxytol nanoparticles, which show a significant correlation between T2* MR hyperintensity and TAM densities [11]. The M1/M2 macrophage paradigm was proposed as a model of intratumoral macrophage heterogeneity and first served to segregate macrophage populations with either pro- or anti-tumor functions. Within this premise, the balance toward M2 macrophages, rather than the total number of TAMs, has been associated with T lymphocyte suppression and may confer a poorer prognosis in some pediatric sarcomas like EWS [12, 13]. In OS, stressed mesenchymal stem cells in the TME have been found to release IL-6 promoting tumor cell survival and predisposing a regulatory microenvironment with recruitment of TAMs that promote wound healing [14, 15]. As cancer immunology continues to expand from TILs to myeloid subpopulations, interrogating their role in pediatric sarcomas will be vital as the TME is likely maintained and shielded by these discrete cell populations.

### Unique stroma of pediatric bone sarcomas

Bony matrix remodeling is a unique feature of pediatric bone sarcomas contributing to the significant genetic and TME heterogeneity that new treatments must overcome [16, 17]. One of the prominent mediators of this remodeling is the receptor activator NFκB ligand (RANKL), RANK, and osteoprotegerin (OPG) system [18]. An increase in RANKL-RANK signaling leads to the development of osteoclasts, triggering bony destruction and growth factor release, in turn stimulating further oncogenic growth. As such, high levels of RANKL have been shown to correlate with osteosarcoma invasion [19]. Locoregional invasion may induce inflammation; elucidation of possible interactions between inflammatory cells (i.e., macrophages) and osteoclasts may shed new light on metastatic biology of these sarcomas. Of note, osteoclasts may play less of a pro-tumoral role in the later course of disease, as their loss may allow for cancer propagation to distal areas such as the lungs [20].

## Checkpoint inhibitors

The 2018 Nobel Prize in Medicine was jointly awarded to James Allison and Tasuku Honjo for their work identifying and characterizing the immunosuppressive function of CTLA-4 and PD-1, respectively. These two proteins belong to an expanding family of molecules known as immune checkpoints, responsible for attenuating the activation and function of their target immune populations. Recent work has begun to elucidate the distinct molecular mechanisms and environmental influences by which CTLA-4 and PD-1 blockade act to enhance anti-tumor immunity [21]. For example, blockade of either the PD-1/PD-L1 or CTLA-4 axis results in expansion of distinct populations of otherwise exhausted intratumoral CD8+ T cells; CTLA-4 blockade also induces expansion of ICOS+ Th1-like cells [21]. Transcriptomic analysis of CD8+ T cells populations from melanoma patients treated with immune checkpoint inhibitors has also identified the transcription factor TCF7 as predictive of positive response to checkpoint inhibitors and could be employed as inclusion criteria for future pediatric sarcoma immunotherapy trials [22].

### PD-1/PD-L1 expression

In addition to the distinct cell populations present within tumors, immune checkpoint expression on myeloid and tumor cells can suppress anti-tumor immunity. For instance, the regulatory ligand PD-L1 on the surface of antigen presenting cells and tumors cells can engage PD-1 on activated T lymphocytes effectively suppressing their function [17]. The PD-L1/PD-1 interaction is associated with downstream signaling inhibition that leads to reduced T cell proliferation and increased T cell apoptosis. In OS, PD-L1 expression correlates with immune cell infiltration, including NK cells, T cells, and dendritic cells, as well as significantly poorer 5-year event free survival [17]. Although PD-L1 blockade has shown effect in various cancers, Majzner et al. recently characterized the frequency of PD-L1 expression in several sarcomas and reported only minimal expression on tumor cells of OS, EWS, and RMS samples [23, 24]. Despite these data, PD-L1 expression on tumor cells may not be necessary for immunotherapeutic effect from immune checkpoint blockade (ICB) [25]; moreover, the role of microRNA may be equally important [26]. MicroRNA-140 in the OS microenvironment has been shown to decrease PD-L1 expression, myeloid-derived suppressor cells, and regulatory T cells and increase CD8^+^ lymphocytes [26]. As better immune correlates for response to immunotherapy are uncovered, the relevance of checkpoint expression, or lack thereof, may be preferentially exploited.

### Immune checkpoint blockade trials in pediatric sarcomas

Unfortunately, the application of ICB toward the treatment of pediatric cancers, including sarcomas, has been very limited. An early trial hypothesized that the expression of cancer testis antigens (CTAs) in adult synovial sarcoma (SS) patients, including the well-studied CTA NY-ESO-1, may render this disease amendable to CTLA-4 blockade with ipilimumab [27]. Unfortunately, the trial was halted early due to concerns regarding toxicity and a lack of clinical activity. Only a couple of clinical trials have been published since on the use of ICB specifically for sarcoma patients [28, 29]. In 2016, the first trial of ipilimumab in children and adolescents ages 2–21 with advanced refractory solid tumors was published (NCT01445379), including 17 patients with a diagnosis of sarcoma [28]. No objective responses were noted; however, of the six patients reported to have stable disease (through 4–10 cycles of drug administration), several were sarcomas (OS, SS, and clear cell sarcoma). Due to the small sample size, it remains difficult to derive any estimate of treatment benefit. Of importance, the number of immune-related adverse events (irAEs) correlated with improved overall survival, suggesting that breaking immune tolerance may be necessary for maximal benefit of ICB in this setting.

A second study (SARC028) seeking to evaluate the efficacy of PD-1 inhibition with pembrolizumab in the setting of bone and soft tissue sarcomas was published in 2017 [29]. In this trial, 18% of patients with soft tissue sarcomas (STS) (including one out of 10 patients with SS), and 5% of patients with bone sarcoma (including one OS and one chondrosarcoma), were determined to have an objective response. No responses were noted in EWS. Subset analysis revealed particularly encouraging results in two types of adult sarcoma—undifferentiated pleomorphic sarcoma and dedifferentiated liposarcoma—leading to an expansion of these cohorts to confirm activity. These results underscore distinctions between pediatric and adult sarcomas (i.e., increased neoantigen burden) highlighted earlier. Finally, a third trial (NCT02500797) evaluating the combination CTLA-4 and PD-1 blockade in metastatic sarcoma patients affirmed the limited efficacy of PD-1 monotherapy, while revealing more promising results in the combination group (5% vs 16% overall response rate, respectively) [30].

### Immune-related adverse events

While reduction of long-term side effects compared to traditional chemotherapy remains a potential benefit of immunotherapy, checkpoint inhibition remains antigen agnostic. This presents risk of irAEs that can manifest as mild-to-severe skin pathologies, endocrinopathies, hepatotoxicity, colitis, pneumonitis, carditis, renal dysfunction, and hypophysitis. Management varies depending on the specific toxicity but can include adjustment of timing and frequency of the immunotherapeutic [31]. Steroids or other immunosuppressants are used when high-grade toxicities are seen, but these may compromise the effectiveness of immunotherapy [32]. Targeted IL-6 blockade can temporize cytokine release syndrome related to PD-1 blockade or T cell therapy, particularly in settings of steroid refractory irAEs [33]. Ultimately, a deeper understanding of mechanisms driving toxicity and cancer-antigen immunotolerance will influence the development of next-generation immunotherapies that balance auto-reactivity and anti-tumor immunity.

### Predicting responses to checkpoint blockade

Despite occasional impressive responses to ICB, only a minority of patients develop avid anti-tumor immunity and impressive clinical responses. Therefore, a major focus of immune checkpoint research has been on identifying predictive biomarkers of response both pre- and on-therapy. Investigations into possible explanations for why patients might fail to initiate or maintain an effective anti-tumor immune response following ICB are ongoing. Emphasis has been placed on tumor mutational burden and PD-1/PD-L1 expression both within the TME and in the periphery, both seemingly predictive biomarkers of response in melanoma. In general, childhood cancers have been shown to possess few mutations and pediatric sarcomas follow this paradigm. Pediatric sarcomas have a low mutational burden, few high-affinity neoepitopes, and fewer expressed neoepitopes, possibly explaining the relative ineffectiveness of checkpoint blockade in these diseases [34, 35]. Furthermore, EWS has little PD-L1 on tumor cells, and in STS including SS, a relative scarcity of tumor-infiltrating T cells and low PD-1/PD-L1 expression may explain a lack of ICB response [36, 37]. Additional studies are beginning to look beyond just single predictive biomarkers; instead, they are incorporating multi-factor analyses that may predict responses better than tumor mutational burden and/or PD-1/PD-L1 expression alone [38].

## Promising immunotherapeutic targets in pediatric sarcoma

To maximize therapeutic effect for pediatric sarcoma patients, methods of altering the sarcoma TME and identification of novel sarcoma antigens should be prioritized (Table 1). While successful immunotherapy is associated with high tumor mutational burdens, few neoantigens (neoAgs) are detected in pediatric cancers [35, 68, 69]. A lack of targetable neoantigens reduces the chance of initiating antigen-specific immune responses following ICB treatment. This is especially relevant for pediatric sarcomas (i.e., OS), which average little more than 7 total neoepitopes per tumor, of which less than half are expressed; however, due to high frequency of oncogenic sarcoma fusion events, fusion-derived neoAgs may serve as alternative and potent tumor-specific targets [35].

**Table 1:** Promising Tumor Microenvironment and Antigen Targets in Pediatric Sarcomas

	Target	Approach	Citations
Tumor microenvironment	Tumor-associated macrophages (TAMs)	Stratification, depletion	[6, 11]
	Immune checkpoints	Checkpoint inhibitors, stratification	[17, 22–30]
	Regulatory T cells	Chemotherapy (preconditioning found to promote anti-tumor immunity, possibly through reduction of regulatory immune populations)	[7, 86]
Sarcoma antigens	Neoantigens	Checkpoint inhibitors, vaccines, combination with PARPi	[35, 39, 68, 70]
	Fusion-derived NeoAgs (e.g. EWSR1-FLI1)	Cancer vaccines, mithramycin, LSD1, lurbinectedin	[35, 44, 46, 47, 50, 78]
	Cancer testis antigens (e.g. NY-ESO-1, MAGE family)	Cancer vaccines, tgTCR-T cells, CAR-T	[52, 45, 49, 79]
	Differentiation antigens		
	Gangliosides (GD2/GD3)	Immunotoxins, CAR-T, CAR-NK	[53, 56, 57, 89, 90]
	HER2	CAR-T	[55, 61, 87]
	CD99	possibly CAR-T	[62]
	CD248	possibly CAR-T	[12, 42, 43]
	IGF1R	Ganitumab	[64–66]
	uPAR/EGFR	Bispecific immunotoxin	[67]

Overexpressed surface targets such as gangliosides GD2 and GD3 are expressed by many tumors, including OS, EWS, and rhabdomyosarcoma. Antibody-based therapy targeting these antigens has shown preclinical promise, with studies ongoing [53]. CD99 is another cell surface protein that is frequently expressed in EWS and occasionally in other sarcomas that may also be amenable to targeting [62]. Alternatively, overexpressed surface targets that drive sarcoma biology (i.e., insulin-like growth factor type 1 receptor (IGF1R), EGFR) may provide additional candidates for a cancer vaccine or T cell therapy (i.e., CAR T cell) [63]. IGF1R has been suggested as a driver of tumor growth in sarcomas [64], and ganitumab, an IGF1R antibody, is currently being studied in trials for metastatic EWS and advanced sarcomas [65, 66]. Expression of urokinase-type plasminogen activator receptor (uPAR) and epidermal growth factor receptor (EGFR) has been shown in sarcoma, with uPAR expressed in EWS and both EGFR and uPAR expressed in RMS [67]. A bispecific ligand targeting both uPAR and EGFR has undergone preclinical testing in mice and canines with promising efficacy, particularly in tumors containing both receptors, and may be amenable for development of bispecific CAR T cells [67]. Finally, CD248/endosialin/TEM1 is present in a variety of tumor environments, with little expression on normal tissues, and has shown potential as a sarcoma target in early studies [12, 42, 43].

Other possible immunotherapeutic candidates include poly ADP ribose polymerase (PARP). PARPs are a group of enzymes found in EWS and STS and their inhibitors could be considered immunotherapeutic sensitizers given their proclivity for inducing DNA damage, which may heighten neoantigen burden [39, 70]. Since pediatric sarcomas are not as immunogenic as their adult counterparts, utilizing combination therapies to unlock immunotherapeutic response may be particularly vital for these malignancies.

## Combination therapies

To break tolerance against self or overexpressed antigens, combination therapies may be necessary. Perturbation of regulatory M2 macrophages with liposomal clodronate in preclinical models of EWS was shown to synergize with oncolytic viral therapy [71]. Additionally, co-delivery of immune activating agonists, such as interleukin-2 (IL-2), in pediatric OS may serve as a necessary adjuvant for targeting overexpressed antigens [44, 51, 72, 73]. While immunotherapeutic platforms can be developed against overexpressed targets, there exists significant risk of autoimmune response as many of these candidates are ubiquitously expressed on normal tissue. For these reasons, expression of mutations and re-expressed developmental antigens (i.e., CTAs) may be more attractive immunotherapeutic targets [45].

Although (as discussed) the number of mutations is limited, some sarcomas like Ewing’s have conserved EWSR1-FLI1 fusions (present in 85% of patients) that may be amenable to immunotherapeutic targeting. Currently, this fusion is being targeted by mithramycin [46, 47], and its analogs EC8042 and EC8105 [48]. Other promising EWS-FLI1 inhibitors include lysine-specific demethylase 1 (LSD1), a demethylating protein that interacts with EWS-FLI1 to mitigate tumor suppression [74], and lurbinectedin, a trabectedin analog that inactivates EWS-FLI1 by redistributing its oncoprotein within the nucleus, that has shown preclinical efficacy in murine models, particularly in combination with the cytotoxic alkaloid irinotecan [75]. As novel immunotherapeutic targets are identified, new platforms for cancer vaccines and adoptive cellular therapy can be co-opted to induce long-lived immunologic response against pediatric sarcomas.

## Cancer vaccines

### Dendritic cell vaccines

Passive immunization can work to prevent infectious disease as observed in maternal transfer of IgG antibodies in utero, or with intravenous gamma-globlin infusion to immunocompromised patients. Passive immunization against cancer can be achieved through directed development of monoclonal antibodies manufactured against surface molecules (i.e., anti-CD20 monoclonal antibodies, rituximab). Often, passive immunization does not confer long-term immunologic memory. Alternatively, active cancer vaccines initiate a local inflammatory response against cancer antigens, which can mediate an antigen-specific T cell response. These T cells can become effector or central memory cells to survey and confer protection against residual tumors or minimal residual disease states. Early reports of novel vaccination approaches in pediatric sarcomas suggest some evidence of antitumor activity.

One of the most common vaccination approaches for pediatric sarcomas is dendritic cell (DC) vaccines. There are generally two approaches to DC vaccination. In the first approach, DCs are pulsed with peptides encoding for antigens that are known to be expressed on the tumor surface. This approach is particularly effective for tumors with known antigenic targets. In the second approach, DCs are pulsed with tumor lysate. This approach bypasses the need for identification of tumor antigens and instead enables DCs to mount responses to any peptide present in the lysate. These approaches are often complemented by inclusion of a known immunogen like keyhole limpet hemocyanin (KLH) to amplify in vivo response.

Early evaluation of DC vaccines for pediatric sarcomas has included both approaches. A phase I study in pediatric solid-tumor patients demonstrated feasibility for generating sufficient DCs from heavily pretreated pediatric patients and some immune responses in patients treated with autologous DCs pulsed with tumor cell lysates [76]. This vaccine produced one partial response in a patient with fibrosarcoma and stable disease in one individual with inflammatory myofibroblastic sarcoma, but failed to prevent progressive disease in patients with OS, EWS, hepatic sarcoma, or desmoplastic round cell tumors [76]. Although a small study of a similar vaccine approach with tumor-lysate pulsed DCs later failed to produce tumor-specific T cell responses in patients with relapsed OS [49], a larger study demonstrated encouraging survival responses for EWS and RMS [77]. In the latter study, 29 pediatric patients with sarcomas were treated with autologous lymphocytes and DCs pulsed with tumor lysate. Treated patients with newly diagnosed EWS and RMS maintained a 77% 5-year overall survival that correlated strongly with the generation of antigen-specific T cell responses [77]. Despite the absence of control arm, this significant correlation between immunologic response and survival supports further evaluation of this therapy.

A second early study evaluated peptide-pulsed DCs. Mackall et al. describe treatment of 30 patients with EWS or alveolar RMS with autologous T cells and DCs pulsed with a peptide sequence generated from the breakpoint region identified for each tumor (EF-1 (EWS/FLI-1) or EF-2 (EWS/FLI-2) for EWS and PXFK (PAX3/FKHR) for alveolar rhabdomyosarcoma) [78]. Although influenza responses were found in all patients, responses to translocation breakpoints were identified in only 39% of patients. Treated patients demonstrated extended overall survival compared to those who did not receive treatment. More recent studies with peptide-pulsed DCs have utilized CTAs. In contrast to the neoantigens introduced at breakpoint regions, CTAs have some level of expression in the body and therefore may require T cells to overcome central tolerance. In a phase I study in patients with relapsed neuroblastomas and sarcomas, DCs pulsed with overlapping peptide pools generated from the full-length CTAs MAGE-A1, MAGE-A3, and NY-ESO-1 produced some T cell responses [79].

### Autologous tumor cell vaccines

In contrast to DC vaccines, which require isolation from peripheral blood and ex vivo antigen pulsing, autologous tumor cell vaccines seek to initiate DC responses in vivo. In this approach, tumor cells are isolated from the patient, pulsed with GM-CSF, and irradiated before being reinjected intradermally or subcutaneously. Recent studies in patients with EWS receiving intradermal tumor cells pulsed with both GM-CSF and an shRNA targeting furin convertase (decreases expression of TGF-β) demonstrated immune responses in 9/18 patients that correlated with survival [80]. The survival was extended in patients receiving the vaccine compared to the patients in a contemporaneous control group [81]. Other trials have been pursued using GM-CSF-secreting vaccines in alveolar sarcoma and clear cell sarcomas but failed to produce tumor regressions [82]. However, post-treatment biopsies revealed an increase in the presence of PD-1+ CD8 T cells with PD-L1+ sarcoma cells [82].

Although formal studies are necessary to draw conclusions, some of the most profound individual responses have been achieved with combination therapies. Tumor lysate-pulsed DCs in combination with peripheral blood stem cell transplantation demonstrated temporary control for a patient with refractory synovial sarcoma and an impressive complete response in a patient with refractory EWS [50]. Similarly, combination therapy with DC vaccines and immune cells (i.e. NK cells) produced an impressive regression of lung metastases in a 9-year-old patient with undifferentiated embryonal sarcoma of the liver [83].

### Non-cell-based vaccines

Similar to autologous tumor cell vaccines, peptide- or viral-based vaccines seek to deliver antigen directly to DCs in vivo. Although evaluation of peptide vaccines has been limited, early trials targeting the breakpoint region of fusion proteins demonstrated immunologic response in only 1/16 patients and mixed clinical response in one patient [84]. Viral vaccines have demonstrated promise in early trials, including one patient with an impressive regression of metastatic recurrent SS after treatment with a DC-targeted lentivirus LV305 [52, 58]. This vaccine is also being evaluated in combination with an NY-ESO-1 peptide and TLR-4 agonist [40]. Other vaccine strategies targeting HER2 have demonstrated benefits in preclinical models, including canine studies of OS in which HER2-targeted listeria reduced the risk of metastasis and improved survival compared to historical controls [41].

Another non-cell-based approach involves direct intratumoral injection of attenuated oncolytic viruses that propagate preferentially in tumor cells that lack innate defenses. Once injected into tumor cells, oncolytic viruses mediate inflammation and a secondary immune response in a similar manner to traditional vaccines. This can lead to tumor lysis and broadening of immunologic response through epitope spreading. Oncolytic HSV1716 has been studied in a recently completed phase I clinical trial (NCT00931931) for relapsed tumors (OS, EWS, STS, and RMS), which may pave the way for new oncolytic viral designs and combinatorial approaches.

## Adoptive cellular therapy

While ICB intends to reinvigorate a suppressed or suboptimal immune response, cancer vaccines seek to induce tumor immune responses through antigen presentation and priming/expansion of new T cell responses. In contrast, adoptive cellular therapy (ACT) circumvents both T cell activation steps targeted by ICB and vaccine approaches; instead, ACT utilizes the direct administration of CD8+ cytotoxic T lymphocytes (CTLs). Early approaches, pioneered by Steven Rosenberg and others at the National Cancer Institute involved the isolation, ex vivo expansion, and infusion of autologous tumor-infiltrating lymphocytes (TILs) into patients with metastatic melanoma [85]. This early work demonstrated impressive clinical responses with ex vivo-manufactured cellular therapies targeting tumor antigens and, highlighted the importance of pre-treatment conditioning to enhance engraftment and persistence of transferred cell populations [85, 86].

Two varieties of ACT have since been developed as feasible alternatives to TIL therapy—transgenic T cell receptors (tgTCR) and chimeric antigen receptors (CAR); both approaches utilize cells engineered to express receptors with known tumor antigen recognition. During an endogenous immune response, self-antigens are presented on class-I MHC to induce CD8+ cytotoxic T cell (CTL) responses, while MHC class-II antigen presentation drives CD4+ helper T cell responses. The presentation of endogenous antigen on MHC-I, including tumor-specific antigens encoded by mutations in intracellular proteins, enables the recognition of virtually any peptide, endogenous or exogenous, by CTLs in an MHC-restricted manner. In contrast, CAR receptors are engineered using an extracellular binding domain from an antibody fused to the intracellular signaling domains of a T cell receptor. This enables high-affinity recognition of only surface antigens due to the nature of antibodies (i.e., no requirement for presentation on MHC) best exemplified by CD19-targeted therapy in hematological malignancies [59, 60]. CAR based approaches have been tried specifically for pediatric sarcomas and include HER2/GD2 CAR T or NK cells.

Osteosarcomas express HER2 at low levels, rendering them resistant to killing by antibody-based approaches but susceptible to killing by HER2 CARs, as shown previously both in vitro and in patient-derived xenograft models [87]. In 2015, a phase I/II trial (NCT00902044) was published evaluating the safety and efficacy of HER2 CARs in 19 patients diagnosed with HER2-positive sarcomas: 16 OSs, one EWS, one primitive neuroectodermal tumor, and one desmoplastic small round cell tumor [61]. No dose-limited toxicities were noted in the study. Although there is one report of a severe adverse reaction following administration of a HER2-CAR to a patient with metastatic colon cancer [54], it was proposed that the lower dose of CAR, utilization of different antigen recognition and signaling domains, lack of IL-2, and no preconditioning contributed to the improved tolerability [61]. Notably, three patients remain in remission beyond 1 year with one patient exhibiting marked necrosis (> 90%) within the tumor following CAR therapy [61]. It remains to be determined whether enhancing in vivo expansion of transfused HER2 CARs will result in improved responses [61].

The gangliosides GD2 and GD3 have been reported to be highly expressed on OS including at recurrence [53, 56, 88], making them attractive targets. In other pediatric sarcomas, it appears GD3 expression remains higher than GD2 [53]. GD2-targeted CARs have shown impressive preclinical activity in H3-K27 M+ mutant gliomas [57] and some anti-tumor activity in EWS models treated with an inducible CAR molecule [89]. Additional preclinical data indicates that transduction of activated NK cells with a GD2-specific CAR can enhance the ability of NK cells to lyse EWS cells in vitro [90]. The preponderance of data across these various studies validates the activity of ACT for pediatric sarcoma patients. A better prospective understanding of who is most likely to respond to will be necessary to maximize ACT responses.

### Conclusion

Although adult cancers such as melanoma and non-small cell lung cancer have responded to immunotherapy, non-specific immunotherapies such as checkpoint blockers may more easily “rescue” an immune response in these patients. Mutations in adult malignancies resulting from missense changes in cancer cells lead to new epitopes that sensitize endogenous immunity for rescue by immune checkpoint inhibitors. Alternatively, most pediatric sarcomas are mutationally bland making endogenous immune responses that can be rescued by immune checkpoint inhibitors more unlikely. However, there may be other avenues to unlock immunotherapeutic response. Overexpressed targets that drive sarcoma biology, fusion transcripts, and developmental antigens such as CTAs may provide alternative avenues for inducing immunity. Bone tumors like OS undergo chromothripsis, which may lead to new breakpoints for immunotherapeutic targeting. Differentiation antigens such as gangliosides are promising targets in pediatric solid tumors such as neuroblastoma and may be co-opted for response against osteosarcoma and Ewing’s sarcoma. Ultimately, de novo induction of immune responses may be requisite in the preponderance of pediatric sarcomas before new agents such as immune checkpoint blockers can be allowed to fully take effect. To achieve this, a better understanding of the tumor microenvironment in pediatric sarcomas may be vital to unlock immunotherapeutic activity. Anecdotal evidence of significant immunotherapeutic response in select patients warrants identification of correlates to better design trials and/or identify patients most likely to respond. These advances juxtaposed to new platform technologies promise to usher new therapies for pediatric patients with refractory sarcomas.

## Data Availability

N/A

## Abbreviations

ACT: Adoptive cell therapy
ALC: Absolute lymphocyte count
CAR: Chimeric antigen receptor
CDK: Cyclin-dependent kinase
CTA: Cancer testis antigen
CTL: Cytotoxic T cell
CTLA-4: Cytotoxic T-lymphocyte-associated protein
DC: Dendritic cell
EGFR: Epidermal growth factor receptor
EWS: Ewing Sarcoma
FGFR: Fibroblast growth factor
GM-CSF: Granulocyte-macrophage colony-stimulating factor
HER2: Human epidermal growth factor receptor 2
HLA: Human leukocyte antigen
ICB: Immune checkpoint blockade
IGF1R: Insulin-like growth factor type 1 receptor
IL-2: Interleukin-2
irAEs: Immune-related adverse events
KLH: Keyhole limpet hemocyanin
MAGE: Melanoma-associated antigen
MDSCs: Myeloid-derived suppressor cells
neoAgs: Neoantigens
NK cells: Natural killer cells
NLR: Neutrophil-to-lymphocyte ratio
OPG: Osteoprotegerin
OS: Osteosarcoma
PARP: Poly ADP ribose polymerase
PARPi: Poly ADP ribose polymerase inhibitor
PD-1: Programmed death-1
PD-L1: Programmed death-ligand 1
RANK: Receptor activator NFκB
RANKL: Receptor activator NFκB Ligand
RMS: Rhabdomyosarcoma
shRNA: Short hairpin RNA
SS: Synovial sarcoma
StS: Soft tissue sarcoma
tgTCRs: Transgenic T cell receptors
TAMs: Tumor-associated macrophages
TEM1: Tumor endothelial marker 1
TME: Tumor microenvironment
TILs: Tumor-infiltrating lymphocytes
Tregs: Regulatory T cells
uPAR: Urokinase-type plasminogen activator receptor
VEGF: Vascular endothelial growth factor
